# Assessment of Standardized Care Plans for People with Chronic Diseases in Primary Care Settings

**DOI:** 10.3390/nursrep14020062

**Published:** 2024-03-29

**Authors:** Glòria Reig-Garcia, David Cámara-Liebana, Rosa Suñer-Soler, Eva Pau-Perich, Miquel Sitjar-Suñer, Susana Mantas-Jiménez, Marta Roqueta-Vall-llosera, Maria del Carmen Malagón-Aguilera

**Affiliations:** 1 Health and Health Care Research Group, Department of Nursing, University of Girona, 17003 Girona, Spain; gloria.reig@udg.edu (G.R.-G.); rosa.sunyer@udg.edu (R.S.-S.); susana.mantas@udg.edu (S.M.-J.); carme.malagon@udg.edu (M.d.C.M.-A.); 2 Department of Nursing, University of Girona, 17003 Girona, Spain; miquel.sitjar@udg.edu (M.S.-S.); marta.roqueta@udg.edu (M.R.-V.-l.); 3 ABS Cassà de la Selva, Institut d’Assistència Sanitària, 17244 Cassà de la Selva, Spain; eva.pau.ias@gencat.cat

**Keywords:** chronic care, standardized nursing care plans, quality of care, nurse management

## Abstract

Background: Aging populations are driving a shift in emphasis toward enhancing chronic disease care, reflected in Catalonia’s regional plan which prioritizes standardized nursing care plans in primary care settings. To achieve this, the ARES-AP program was established with a focus on harmonizing standards and supporting routine nursing clinical decision-making. This study evaluates nurses’ perceptions of ARES-AP’s standardized care plans for chronic diseases. Methods: A mixed-methods approach based on an ad hoc questionnaire (n = 141) and a focus group (n = 14) was used. Quantitative data were statistically analysed, setting significance at *p* < 0.05. Qualitative data were explored via content analysis. Results: ARES-AP training was assessed positively. The resources for motivational interviewing and care plans for the most prevalent chronic diseases were rated very positively. This study identified key factors influencing program implementation, including facilitators such as structured information and nursing autonomy, barriers such as resistance to change, motivators such as managerial support, and suggested improvements such as technological improvements and time management strategies. Conclusions: This study identifies areas for improvement in implementing standardized nursing care plans, including additional time, motivation, enhanced IT infrastructure, and collaboration among primary care professionals. It enhances understanding of these plans in primary care, especially in managing chronic diseases in aging populations. Further research should assess the program’s long-term impact on chronic patients. This study was not registered.

## 1. Introduction

As the population ages across European countries, there is a parallel increase in the prevalence of chronic diseases. In the European Union, approximately one-third of individuals reported long-standing health issues in 2021, with an notable portion grappling with multiple chronic conditions [1,2,3,4]. This demographic shift has led to a heightened demand for healthcare services [5], which often means that care is shared by different professionals, providers, and areas. Therefore, ensuring the quality and safety of continuity care needs could be met through personalized nursing care plans [6,7].

The significance of personalized care planning for delivering high-quality, patient-centred care is underscored by various approaches. Despite variations in content and terminology, these approaches share common elements, emphasizing patient–provider discussions, the development of holistic plans addressing clinical and non-clinical needs, and mechanisms for sharing plans among providers to coordinate care [8,9,10]. Individualized care plans involve tailoring standardized approaches to meet the unique needs and goals of each patient, offering personalized and holistic care and serving as pre-established guides, ensuring consistent care for patients with specific conditions and streamlining tasks to eliminate repetitive activities [11]. Implementing standardized care plans serves as a method of standardizing documentation structures, enhancing the quality of record content [12]. Moreover, these plans have demonstrated effectiveness in managing chronic diseases [13,14].

### Standardized Nursing Care Plans in Catalonia Primary Care Settings

Catalonia, situated in northeastern Spain, faces the challenges posed by a significantly aged population [15,16]. In response, its regional strategic plan prioritizes enhancing care for chronic conditions, aiming to promote health and reduce risks associated with impactful chronic diseases [7,17,18]. The plan involves the introduction of standardized nursing care plans to primary care settings. To achieve this, the ARES-AP program was established with a focus on harmonizing standards and supporting routine nursing clinical decision-making, specifically in the context of chronic conditions [19,20]. The ARES-AP program is grounded in the nursing interface vocabulary, ATIC (Architecture, Terminology, Information, Interface, Nursing and Knowledge), implemented in the electronic health Catalan records system. ATIC provides a concept-oriented, interface-controlled vocabulary for assessing patients’ health, problems, circumstances, and nursing interventions. Derived from the natural language used by nurses, ATIC has become a reliable nursing terminology interface for crafting care standards [21,22,23].

Over the course of 2 years, the team of territorial clinical nurses in all primary healthcare areas has collaboratively developed and standardized diverse care plans, specifically designed to address the prevalent reasons for visits by primary care nurses, particularly for individuals with chronic conditions. Each plan includes crucial resources such as tools for conducting motivational interviewing, health education, therapeutic planning, and assessments of social dimensions [24].

Recent research emphasizes the need to improve nursing documentation in the healthcare sector [25]. Therefore, evaluating the quality of standardized nursing care plans could provide insight into best practices and limitations in order to improve their quality as well as patient outcomes. According to Evat, nurses’ use of standardized care plans was influenced by the plans’ partial implementation, their views on usefulness, and their personal views on the detail required in a care plan. However, most studies on standardized care plans focus on hospital settings [26,27]. The aim of this study is to explore nurses’ perceptions of ARES-AP-standardized nursing care plans for people with chronic diseases in primary care settings.

## 2. Methods

### 2.1. Design

We adopted a mixed-methods approach [28] using a convergent parallel design. In this design, the quantitative and qualitative strands of the research are performed independently, and their results are brought together in the overall interpretation [29].

The quantitative phase, with a cross-sectional design, encompassed data collection and analysis via a custom-designed questionnaire, while the qualitative phase encompassed a focus group discussion to elucidate different perspectives on the ARES-AP individualized standardized care plans for chronic conditions treated in primary care centres [30].

### 2.2. Quantitative Research

All 27 basic health areas in the Girona healthcare region (encompassing 100% of the corresponding primary care centres) were included in this study. The Girona healthcare region covers an area of 5754 km^2^ and provides healthcare to 883,512 inhabitants [31].

The clinical reference nurse for each of the 27 areas, using an email distribution list, invited all nurses operating in that area to participate in the study. Inclusion criteria were working in their primary health centre during the study period and using ARES-AP plans for people with chronic conditions.

Participation consisted of self-completing a short anonymous ad hoc questionnaire consisting of a section covering nurse sociodemographic data and a section recording nurses’ perceptions of ARES-AP. This second section consisted of seven questions related to (1) training; (2) leadership; (3) resources for motivational interviewing, health education, therapeutic planning, social dimensions, and self-care; (4) satisfaction with specific plans for the most prevalent chronic diseases (hypertension, diabetes, chronic obstructive pulmonary disease, cardiac insufficiency, and obesity), for people with complex chronic diseases, for chronic wound care, and for less prevalent chronic diseases (e.g., fibromyalgia); (5) perceived usefulness; (6) barriers to implementation; and (7) proposals for improvement.

Answers to questions 1–5 were measured on a 5-point Likert scale (1 for lowest score to 5 for highest score) and answers to questions 6 and 7 were open-ended. Clinical experts (two territorial ARES-AP reference nurses) and a methodological expert participated in questionnaire development. Before starting the study, the questionnaire was piloted in 25 primary care nurses, resulting in minor modifications. Good internal consistency was confirmed by Cronbach’s alpha values > 0.92.

Statistical data analysis was performed using IBM SPSS AMOS and Statistics version 28 software, with the significant level set to <0.05 for all analyses.

Continuous variables were described in terms of tendency and dispersion measures, namely mean, standard deviation (SD), median, and interquartile range (IQR). Categorical variables were described as absolute frequencies and percentages. Bivariate analysis was performed with the Mann–Whitney U test, Kruskal–Wallis test, and Spearman correlation coefficient.

### 2.3. Qualitative Research

A generic qualitative design [32] based on a constructivist naturalistic approach was used, as it enables understanding of the complexity of a phenomenon from differing points of view of informants [33]. Generic studies allow researchers to play with boundaries, use established methodological tools, and develop research designs that fit their epistemological stance, discipline, and particular research questions [34].

Our qualitative research consisted of a focus group session with clinical reference nurses, whose functions, established in 2020, included leading new patient care programmes (including ARES-AP) in primary care. Focus group participants were selected from different basic health areas using intentional sampling [35], i.e., the researchers selected the participants from among the 27 clinical reference nurses attached to the basic health areas. The homogeneity and heterogeneity criteria were their ability to provide relevant information and their workplace, respectively. The focus group followed a semi-structured format, based on open-ended questions regarding the ARES-AP programme. The questions were carefully crafted based on insights from key stakeholders, including the Girona healthcare region territorial reference nurse for ARES-AP and a nurse with expertise in conducting focus groups, and after thoroughly reviewing evidence regarding standardized nursing care programmes [19,23] and the development of semi-structured interview scripts [36] (Table S1 in the Supplementary Materials).

The focus group discussion was conducted by two researchers, one acting as a moderator and the other taking notes (including of non-verbal communications) and summarizing the discussion for reporting back at the session’s end. The session, which took place in a suitable primary care centre room, lasted around 55 min, and was audio-recorded and later transcribed verbatim.

Two researchers analysed the resulting data using content analysis, defined by Krippendorf [37] as a means of making replicable and valid inferences from text, and involving decontextualization, recontextualization, categorization, and compilation [38]. To ensure the validity of our results, themes were discussed and clarified by the research team until a consensus was reached [39].

### 2.4. Ethical Considerations

The study, carried out in accordance with the Declaration of Helsinki and all relevant regulations and guidelines, was approved by the territorial research ethics committee (Jordi Gol: code 22/121-P). The nurses granted their informed consent to participation, which was voluntary.

## 3. Results

### 3.1. Quantitative Research Results

The final sample included 141 primary care nurses, 95.7% (n = 135) women, mean (SD) age 40.6 (9.4) years. The participants had a mean (SD) of 17.1 (9.6) years’ experience as nurses and 14.4 (8.4) years’ experience as primary care nurses. As for other characteristics, 80.9% (n = 114) were employed on permanent contracts, 88.7% (n = 125) were care nurses, and 54.6% had received ARES-AP training before the COVID-19 pandemic (Table 1).

All management nurses, 90% of case management and aged-care-home management nurses, and 79.2% of care nurses were employed on a permanent contract (*p* < 0.05).

Table 2 summarizes the results for nurse perceptions regarding training, leadership, resources, and their satisfaction with, and usefulness of, the ARES-AP plans for chronic conditions.

The nurses generally perceived having received good ARES-AP training and perceived being satisfied with ARES-AP leadership, with no significant differences observed in any of these variables according to gender (Mann–Whitney U test; *p* > 0.05), employment relationship (Mann–Whitney U test; *p* > 0.05), role, or the time of training (Mann–Whitney U test; *p* > 0.05).

Regarding ARES-AP resources, the highest- and lowest-scored resources were, respectively, motivational interviewing and social dimensions. Perceptions of ARES-AP resources assigned to health education showed significant differences depending on nursing roles, with case management nurses having higher scores (case management nurses, mean 3.83; management nurses, mean 3.70; and care nurses, mean 3.13; Kruskal–Wallis test; *p* = 0.04). No differences were observed in resource scores according to gender (Mann–Whitney U test; *p* > 0.05), age (Spearman’s correlation; *p* > 0.05), time of training (Mann–Whitney U test; *p* > 0.05), employment relationship (Kruskal–Wallis test; *p* > 0.05), or experience as a primary care nurse (Spearman’s correlation; *p* > 0.05).

Satisfaction scores for ARES-AP plans were highest for the most prevalent chronic conditions and lowest for less prevalent chronic conditions. Moreover, moderate satisfaction was observed in the ARES AP plans for complex chronic conditions and for chronic wound care. Nurses’ satisfaction with the ARES-AP plans for the most prevalent chronic diseases differed according to the nurse’s role. In particular, greater satisfaction with these care plans were expressed by nurses working in management positions (Kruskal–Wallis test; *p* = 0.04). Any of the other ARES-AP plans showed significant differences according to the rest of the sociodemographic characteristics.

Most of the nurses perceived ARES-AP plans to be especially useful for the most prevalent chronic diseases and for chronic wounds. ARES-AP plans for the most prevalent chronic diseases were identified as more useful for nurses with temporary employment relationships (Mann–Whitney U test; *p* = 0.02). There were no statistically significant differences for other ARES-AP usefulness dimensions (*p* > 0.05).

Table 3 summarizes the details of the main barriers to implementing ARES-AP plans and proposals for improvements, as noted in questionnaire responses to open-ended questions. The main barriers identified were a lack of training and of experience and knowledge of the ARES-AP programme, and also issues related to ARES-AP programme functioning. Proposals for improvement included regular training, involvement of all primary healthcare providers, and enhancement of certain care plan aspects.

### 3.2. Qualitative Research Results

The focus group discussion was conducted with 14 clinical reference nurses aged 36–58 years working in different primary care centres. Table 4 summarizes content analysis results, reflecting four themes related to ARES-AP: (1) facilitators; (2) barriers; (3) motivations; and (4) suggested improvements.

**Topic 1. Facilitators**

The clinical reference nurses considered that ARES-AP helped organize information, was a guarantee of safety, increased nurse visibility and autonomy, and had positive programme-specific features. Most nurses believed that the ARES-AP plan structure greatly facilitated the organization of information and so enhanced nursing care and tracking, with the individualized plans offering a clearer picture of care journey stages.

*“It is a way of organizing the information we have on the patient”*

[P2]

Regarding safety, the flexibility to customize plans was highlighted, as this meant they could be adapted to the needs and circumstances of each patient.

*“They are not closed plans, so you can make modifications, introduce variables that it is important to assess… they are flexible and can be shaped to the needs of the patient”*

[P4]

Additionally, the fact that the ARES-AP plans incorporate evidence-based guidelines also helped nurses work in a more objective and secure manner, especially when patients with chronic diseases sought urgent consultations.

*“ARES-AP enhances safety in urgent cases, because since everything is protocolized and based on guidelines, you know you haven’t forgotten anything”*

[P6]

Regarding records, the nurses stated that ARES-AP enabled swift, visual, and well-structured record-keeping. They highlighted the fact that reduced free-text sections facilitated the visualization of relevant information and reduced the burden of unnecessary data. ARES-AP thus improved the recording of interventions, health education initiatives, warning signs, and follow-up instructions. According to the participants, prior to ARES-AP implementation, many nursing activities were not adequately documented.

*“You see instantly, in a minute, all the monitoring details of our interventions and I think that visually you always can see where you are in monitoring”*

[P1]

*“…it is a way to record all health education provided that is not recorded anywhere else”*

[P5]

This improvement in record-keeping facilitated both interprofessional communication and care continuity in primary care, and according to the participants, was especially beneficial for patients with chronic conditions receiving care in different primary care centres.

*“I find it very useful for patients from other regions who, because of holidays, etc, are in our region” (referring to care continuity).”*

[P7]

The participants emphasized that the ARES-AP programme highlighted the role of nurses in caring for and enhancing the wellbeing of individuals and communities, and also underscored the importance of nursing care for less prevalent health conditions.

*“It makes the work we do more visible.”*

[P1]

As for the research role of nurses, it was felt that the greater ease of retrieval and analysis of data from coded records streamlined through the ARES-AP programme enabled research.

*“We can do research. It used to be all free text and collecting data was extremely complicated.”*

[P3]

Some participants believed that ARES-AP plans were a useful decision-making aid that ultimately ensured greater effectiveness in managing chronic health problems. The simplified record-keeping also left more time for thinking about the customization of plans and the inclusion of new plans.

*“It helps to know what needs to be dealt with first… you open the plans and immediately see what needs to be done.”*

[P11]

Finally, some inherent features of the ARES-AP programme were also considered facilitators, including its usefulness, ease of use, and the possibility of including new protocols and care plans according to individual or community needs. As an aid to nurses in their work, it was considered especially valuable for nurses new to primary care.

*“For new primary care nurses, coming from hospitals, it makes their job easier.”*

[P2]

**Topic 2. Barriers**

The three main barriers to using ARES-AP highlighted by the nurses were resistance to change by some healthcare providers, a lack of ARES-AP integration with routine nursing practice, and a lack of ARES-AP training for nurses.

Participants identified the resistance of some professionals to change as one of the primary barriers to ARES-AP.

*“Colleagues’ resistance to change is an important barrier. Other changes have been made in the past and it is complicated”*

[P6]

This resistance to change was particularly related to the increased workload that required the creation of care plans for patients with chronic health issues; this necessitated additional time in consultations, often not available. Nurses also explained that while patient care in nursing consultations was similar, ARES-AP plans introduced an additional workload in terms of record-keeping. Nursing records were previously mainly based on free text, whereas ARES-AP imposed an information technology (IT) learning curve in relation to predefined screens with different elements. While this perception of overload was particularly evident in nurses who had previously used other platforms, it was not a barrier for new nurses starting out in primary care or community care.

*“Yes, it is true that it has been an effort for people in primary care, but for people starting out it is an advantage”*

[P7]

IT difficulties were identified as another source of resistance to change, as nurses felt that it was often challenging to reach a consensus between nurse and IT department needs regarding ARES-AP use.

*“…IT problems are indeed a difficulty, sometimes it is difficult for IT people to understand you”*

[P4]

Another stated barrier was the lack of ARES-AP integration into routine workflows. Once a consultation was completed, nurses often omitted to include information in the ARES-AP plan. According to the participants, this was because the lack of integration made record-keeping burdensome.

*“If you could record details while you visited it would be much easier”*

[P3]

Lastly, a significant barrier was that ARES-AP training and inclusion in primary care occurred during the difficult period of the COVID-19 pandemic, leaving nurses feeling that training had been less than effective. Participants believed that, without proper training, effectively implementing ARES-AP was challenging.

*“Of course, we received training during COVID but maybe we need to do new training”*

[P3]

**Topic 3. Motivations**

The nurses generally perceived that their colleagues were not very motivated to use ARES-AP plans, especially healthcare providers with more work experience and more primary care experience. In contrast, however, motivation among newer nurses was high.

*Professionals who come in new …. there is a nurse who has already started working in this way … when they finished their degree, ARES-AP was already underway. So they do not see it as strange, they even say that they do not understand why there is so much criticism…”*

[P5]

A motivational factor highlighted for ARES-AP plan use was a lower clinical workload.

*“Those who have more time use it more, I think they are more motivated”*

[P9]

Sources of motivation included receiving periodic ARES-AP training and properly understanding ARES-AP functioning, with the clinical reference nurses underlining the importance of their role as trainers.

*“I do periodic training and the results are good”*

[P4]

The participants believed that their role as clinical reference nurses should serve to motivate other nurses to use ARES-AP plans. However, they indicated that this primary care role was relatively new, so they had not had sufficient time to evaluate their work as ARES-AP leaders.

*“As reference nurses we must constantly motivate them”*

[P8]

The participants all agreed that motivation was significantly higher when there was clear support from primary healthcare team management, with financial incentives for ARES-AP use by nurses proving to be particularly motivational. They also considered that monitoring would improve motivation.

*“It’s clear that when it’s targeted by DPOs [financial incentives], then it’s more interesting for them to use it”*

[P8]

**Topic 4. Suggested improvements**

Some proposed ARES-AP improvements were related to IT aspects, essentially improving the often challenging communications between nurses and IT staff and improving the visualization of ARES-AP records on various screens.

*“IT changes, to make things easier”*

[P1]

Other improvement proposals were to dedicate more time to training teams, designing strategies to promote ARES-AP plan use within teams, and monitoring effectiveness.

*“We need time to train but also time to analyse how the team is doing and to prepare a training strategy”*

[P4]

Differences between the management teams at different centres were noted, with the consensus being that across-the-board support from all management teams was crucial to be able to consolidate the ARES-AP programme in various primary care areas.

*“Depending on the team, it may be easier to organize yourself, to get the time you need, or they understand that you need this time”*

[P6]

Another improvement proposal was to involve all primary care healthcare providers responsible for chronic health issues, not just nurses, in using ARES-AP, i.e., doctors, psychologists, social workers, etc.

*“Now we all need to work on it, few doctors use it, or social workers”*

[P13]

## 4. Discussion

In Catalonia (Spain), individualized and standardized nursing care plans introduced through the ARES-AP program in primary care settings focus on individuals with chronic conditions. This study indicates a positive perception of ARES-AP training among nurses. Satisfaction with ARES-AP varied, with resources for motivational interviewing deemed satisfactory, those for therapeutic planning and managing chronic wounds rated average, and resources for social dimensions and self-care considered poor. The focus group findings emphasize the potential benefits and challenges of implementing ARES-AP in primary care, highlighting the importance of factors such as having enough time for care plan registration, training, nurse motivation, healthcare provider involvement, and continuous IT improvements for successful workflow integration and utilization. This study concludes that nurse perceptions of ARES-AP depend on organizational, professional, and individual factors, aligning with evaluations of other nursing programs [40].

Nurses who received training before the COVID-19 pandemic reported greater satisfaction with both ARES-AP training and leadership; this underscores the difficulties occasioned by the pandemic [41] and the importance of reinforcing training strategies.

ARES-AP resources were rated as especially good for motivational interviewing. While there is no literature on its use in standardized nursing care plans, this resource has been reported in positive terms for patient discharge planning [42]. Our results can therefore be considered positive in view of the large body of literature confirming the effectiveness of motivational interviewing for numerous outcomes and circumstances, including primary care [43,44] and chronic conditions [45]. The fact that social dimension resources were rated less positively would suggest that patients’ social problems tend to be overlooked [46,47], as confirmed by other studies of nursing programmes [6]. Health education resources were rated as moderate, suggesting that further efforts in this area are necessary, given the key role of health education in the community management of chronic diseases [48]. Health education is basically a care nurse function, and the fact that care nurses rated these resources more negatively underlines a corresponding need for improvement. Finally, in relation to resources for self-care and therapeutic planning, our results also point to a need for improvement, given that both aspects are crucial to chronic disease management [49].

Regarding ARES-AP plans for specific conditions, a high level of satisfaction was expressed regarding plans for the main chronic health concerns (hypertension, diabetes, chronic obstructive pulmonary disease, dyslipidaemia, and mental health problems in the Spanish primary care population aged 15 years and older [50]). Satisfaction was lower, however, with plans for less prevalent chronic diseases (e.g., fibromyalgia). Care strategies for individuals with less prevalent chronic diseases need to be reinforced, not only because of the disproportionate societal and healthcare costs, but also because nursing care has been documented to be pivotal in enhancing the quality of life of the affected individuals [51]. Chronic wounds also have a profound impact on quality of life [52], with many health systems including wound management as a patient safety strategy [53]. Corroborating satisfactory results reported for previous programmes to identify, evaluate, and treat chronic wounds [54], the ARES-AP plan for chronic wounds received positive feedback from nurses. Broadly speaking, the fact that the effectiveness of ARES-AP plans seems to be influenced by nurses’ level of experience underscores the value of the plans in supporting clinical practice by less experienced nurses.

This study identified several facilitators of ARES-AP use, including the following: the organized structuring of information, which enhances tracking and understanding of individual care needs; the inclusion of evidence-based guidelines, which ensures safety and customization; improved record-keeping, which fosters interprofessional communication and care continuity; heightened recognition of the vital role of nurses in managing chronic diseases; and greater nurse autonomy in decision-making and care provision. These facilitators underscore the potential benefits of ARES-AP in terms of improved organization, safety, and efficiency, and also in recognizing nursing contributions to chronic care, documented as essential for this patient population [6,55]. Previous studies have reported dissatisfaction with standardized care plan programmes, particularly arising from a lack of consensus as to care plan structure [29]. The positive evaluation of ARES-AP plans by our study participants, however, points to a broad consensus regarding plan structure.

As for barriers to ARES-AP use, these were mainly related to a resistance to change, typically related to increased workloads and IT challenges. Previous studies assessing barriers to the implementation of standardized care plans also point to a resistance to change, as well as poor perceptions of their usefulness [12,56]. Moreover, while IT integration in healthcare is viewed as a sociotechnical issue, some studies of nursing care programmes underline the fact that IT systems fail to adequately support nursing workflows [57]. Additional obstacles were a lack of training and of ARES-AP programme integration in regular workflows. Therefore, crucial to successful ARES-AP adoption in primary care settings is overcoming resistance to change, improving workflow integration, and providing more comprehensive training, all of which may necessitate additional resources and support. Improved training and managerial support have been demonstrated to be effective in the implementation of other programmes among nurses [58].

Our clinical reference nurses perceived motivation to use ARES-AP plans by their colleagues to be low. Studies on standardized care plans in other countries underline the fact that engaged individuals create the necessary culture for successful implementation [59]. According to Janssen [60], nurse motivation and engagement are correlated with factors such as working conditions and quality of supervision. This would suggest that allocating sufficient time and providing effective leadership could successfully overcome the identified barriers to ARES-AP implementation.

Our study participants also attached importance to expanding ARES-AP use to all primary care professionals (including doctors and social workers). This is corroborated by evidence suggesting that partial implementation of health programmes creates an opportunity for non-use [12].

Finally, our results overall are aligned with the 2015 WHO global strategy for people-centred care based on eight principles [61], one of which is integrated care.

## 5. Limitations

Regarding the limitations of our study, although most Catalan primary care centres are public and implement the ARES-AP programme, the corresponding care plans may not be representative of similar plans used in other health systems. Nonetheless, our findings may suggest where general improvements could be made to other care plan programmes for people with chronic diseases.

With regard to the quantitative research, the cross-sectional design limits this study to an analysis of relationships between variables without the possibility of establishing causality. Furthermore, since no validated instrument existed, we were obliged to create an ad hoc questionnaire to collect the data. Nonetheless, our Cronbach alpha values do indicate a good reliability of the questionnaire, which we based on the best available evidence and on expert input (ARES-AP programme clinical reference nurses and methodological expertise). However, further research into validated instruments that evaluate nursing programmes like ARES-AP is required.

The transferability of our results may be limited by our use of a single focus group to examine key aspects of our quantitative research results in depth. However, to ensure as broad a perspective on ARES-AP as possible, the focus group was intentionally sampled from clinical reference nurses leading primary care teams. The perspectives of those participants offered valuable insights into how entire teams perceive and engage with the ARES-AP programme and plans. Our study findings, nonetheless, highlight the need to incorporate other healthcare providers into ARES-AP implementation, so future research will focus on evaluating the perceptions of these other professionals.

## 6. Conclusions

Our study assessed the implementation of ARES-AP standardized nursing care plans for chronic conditions in primary care. Although the nurses expressed their satisfaction with the educational sessions and acknowledged the effectiveness of ARES-AP in providing tools for motivational interviewing, they identified specific areas for improvement. Specifically, there is a need to enhance resources for evaluating social dimensions and addressing less prevalent chronic diseases.

The findings of the focus group highlighted the most significant elements to achieve the most effective execution, such as additional time, thorough training, motivation, an enhanced IT infrastructure, and a cooperative approach encompassing all primary care healthcare professionals. In conclusion, addressing these key issues is crucial for the comprehensive and effective utilization of ARES-AP, providing potential benefits for both healthcare providers and patients. Further investigations should be performed in order to determine the program’s impact on the long-term health outcomes of individuals with chronic diseases.

## Figures and Tables

**Table 1:** Sociodemographic and occupational characteristics of the sample.

	Sample n = 141
**Age** mean (SD) [median] [IQR]	40.6 (9.4) [41] [32.00–47.00]
**Nursing experience** mean (SD) [median] [IQR]	17.1 (9.6) [18.5] [8.50–23.75]
**Primary care nursing experience** mean (SD) [median] [IQR]	14.4 (8.4) [10] [3.25–18.75]
**Gender** mean (SD)	
Women	135 (95.7)
**Employment relationship** mean (SD)	
Permanent contract	114 (80.9)
Temporary contract	27 (19.1)
**Role** mean (SD)	
Care	125 (88.7)
Management	6 (4.2)
Case management or aged-care-home management	10 (7.1)
**ARES-AP training** mean (SD)	
Before COVID-19	77 (54.6)
During COVID-19	64 (45.4)

**Table 2:** Perceptions of ARES-AP.

	Sample n = 141
**Training** (SD); [median] [IQR]	3.25 (2.8) [3.00] [2.00–4.00]
**Leadership** (SD); [median] [IQR]	3.03 (0.9) [3.00] [2.00–4.00]
**Resources** (SD); [median] [IQR]	
Motivational interviewing	3.69 (1.0) [3.00] [2.00–4.00]
Health education	3.21 (1.0) [3.00] [2.00–4.00]
Therapeutic planning	3.17 (0.2) [3.00] [2.00–4.00]
Social dimensions	2.80 (0.9) [3.00] [2.00–4.00]
Self-care	2.90 (0.8) [3.00] [3.00–4.00]
**Satisfaction with ARES-AP** (SD); [median] [IQR]	
Most prevalent chronic diseases	3.69 (1.0) [3.00] [3.00–4.00]
Complex chronic diseases	3.17 (0.9) [3.00] [3.00–4.00]
Chronic wound care	3.21 (1.04) [3.00] [2.00–4.00]
Less prevalent chronic diseases	2.90 (0.8) [3.00] [3.00–4.00]
**Usefulness of ARES-AP** (SD); [median] [IQR]	
Most prevalent chronic diseases	3.36 (1.1) [3.00] [3.00–4.00]
Complex chronic diseases	3.12 (0.85) [3.00] [3.00–4.00]
Chronic wound care	3.21 (1.04) [3.00] [2.00–4.00]
Less prevalent chronic diseases	2.92 (0.84) [3.00] [3.00–4.00]

**Table 3:** Main barriers to ARES-AP implementation and suggested improvements.

Main Barriers	Suggested Improvements
Lack of training	Offer periodic training
Lack of experience	Ensure all professionals working in primary healthcare settings are aware of the programme
Lack of knowledge	Base documentation on fewer screenings and fewer completion steps
Not all primary healthcare providers are users (doctors, psychologists, etc.)	Make care plans more current, visual, and responsive
Not intuitive	Group care plans with similarities together
Documentation is time-consuming	Directly generate diagnoses for health problems
Overly detailed	Improve plans for acute conditions
Too much and/or poorly organized information	Highlight key variables in each plan
Lacking in clarity	Improve plans, e.g., for patients with chronic wounds.
Complicated if dealing with ≥2 chronic diseases	
Data not recorded in clinical histories	

**Table 4:** Content analysis results for themes related to ARES-AP.

Topic 1 Facilitators	Topic 2 Barriers	Topic 3 Motivations	Topic 4 Suggested Improvements
**Information structuring** -Standardized plans -Planning -Monitoring	**Resistance to change** -Additional workload -Additional documentation time -Need for IT support	**Motivation level** -Low	**IT aspects** -Better communication with IT technicians -Delays to IT changes
**Safety** -Flexibility -Guidelines -Evidence-based -Quality records -Interprofessional communication -Care continuity	**Lack of integration in care activities** -Difficult to simultaneously visit and document	**Motivational factors** -Less healthcare pressure -Periodic training -Good programme knowledge -Management support -Incentives -Work monitoring	**Time burden (reference nurses)** -Training time -Team monitoring time
**Nurse visibility** -Enhances nursing role -Enables research	**Lack of training** -More training -Timing of training		**Management team support** -More consistent support
**Nurse autonomy** -Decision-making aid			**Interdisciplinary implementation**
**Programme features** -Useful -Easy to use -Facilitator for new nurses -Updated according to individual/community needs			

IT = information technology.

## Data Availability

Data is in the article.

## Supplementary Materials

The following supporting information can be downloaded at: https://www.mdpi.com/article/10.3390/nursrep14020062/s1, Table S1: Semi-structured ARES-AP focus group guide.

## Public Involvement Statement

The participants engaged by responding to a questionnaire regarding their perceptions of the ARES-AP program. Additionally, some of them took part in a focus group discussion exploring the barriers and facilitators associated with the program’s implementation in nursing standardizing care plans.

## Guidelines and Standards Statement

This manuscript was drafted against The Mixed-Methods Appraisal Tool (MMAT) [62].

## References

[1] Busse R., Blumel M., Scheller-Kreinsen D., Zentner A. (2010). Tackling Chronic Disease in Europe: Strategies, Interventions and Challenges.

[2] Eurostat Statistic Explained Chronic Morbidity: Long-Standing Illenesses or Health Problems. https://ec.europa.eu/eurostat/statistics-explained/index.php?title=Self-perceived_health_statistics&oldid=509628.

[3] Salive M.E. (2013). Multimorbidity in Older Adults. Epidemiol. Rev..

[4] Salisbury C., Johnson L., Purdy S., Valderas J.M., Montgomery A.A. (2011). Epidemiology and impact of multimorbidity in primary care: A retrospective cohort study. Br. J. Gen. Pract..

[5] Wijlhuizen G.J. (2012). Impact of multimorbidity on functioning: Evaluating the ICF core set approach in an empirical study of people with rheumatic diseases. J. Rehabil. Med..

[6] Reig-Garcia G., Bonmatí-Tomàs A., Suñer-Soler R., Malagón-Aguilera M.C., Gelabert-Vilella S., Bosch-Farré C., Mantas-Jimenez S., Juvinyà-Canal D. (2022). Evaluation and perceptions of a nursing discharge plan among nurses from different healthcare settings in Spain. BMC Health Serv. Res..

[7] Generalitat de Catalunya Pla de Salut. https://salutweb.gencat.cat/ca/eldepartament/pla-salut/.

[8] Edwards S.T., Dorr D.A., Landon B.E. (2017). Can Personalized Care PlanningImprove Primary Care?. JAMA.

[9] Baker A., Cronin K., Conway P., DeSalvo K., Rajkumar R., Press M. Makingthe Comprehensive Shared Care Plan a Reality. https://catalyst.nejm.org/doi/full/10.1056/CAT.16.0838.

[10] Sullivan S.S., Mistretta F., Casucci S., Hewner S. (2017). Integrating social context intocomprehensive shared care plans: A scoping review. Nurs. Outlook.

[11] Nursing Care Plan (NCP): Ultimate Guide & List [2024 Update]—Nurseslabs. https://nurseslabs.com/nursing-care-plans/.

[12] Østensen E., Hardiker N.R., Bragstad L.K., Hellesø R. (2020). Introducing standardised care plans as a new recording tool in municipal health care. J. Clin. Nurs..

[13] Bodenheimer T., Wagner E.H., Grumbach K. (2002). Improving primary care for patients with chronic illness—The chronic care model, part 2. JAMA.

[14] Burt J., Rick J., Blakeman T., Protheroe J., Roland M., Bower P. (2014). Care plans and care planning in long-term conditions: A conceptual model. Prim. Health Care Res. Dev..

[15] Institut d’Estadística de Catalunya Idescat Taules de Vida (2015–2019). https://www.idescat.cat/pub/?id=iev.

[16] Institut d’Estadística de Catalunya Idescat Fecunditat. https://www.idescat.cat/pub/?id=inddt&n=917.

[17] Zamora-Sánchez J., Zabaleta-del-Olmo E., Gea-Caballero V., Julián-Rochina I., Pérez-Tortajada G., Amblàs-Novellas J. (2021). Correction to: Convergent and discriminative validity of the frail-VIG index with the EQ-5D-3L in people cared for in primary health care. BMC Geriatr..

[18] Scientia Bones Pràctiques en Altenció Compartida: Recomenacions per a una gestió òptima Dels PIIc. https://scientiasalut.gencat.cat/handle/11351/7010.

[19] Institut Català de la Salut ARES Primària. https://ics.gencat.cat/ca/assistencia/cures-infermeres/cures-atencio-primaria/ares_primaria/index-ap.html.

[20] Juvé-Udina M.E. (2012). Validity Evaluation of an Interface Nursing Terminology. Ph.D. Thesis.

[21] Rios Jimenez A.M., Artigas Lage M., Sancho Gómez M., Blanco Aguilar C., Acedo Anta M., Calvet Tort G., Hermosilla Perez E., Adamuz-Tomás J., Juvé-Udina M.E. (2020). Standardized nursing languages and care plans. Perception of use and utility in primary healthcare. Aten. Primaria.

[22] Juvé Udina E. Atic. http://aticcare.peoplewalking.com/clasificacion-diagnostica/.

[23] Juvé-Udina E. (2016). La Terminologia ATIC: Eje Diagnóstico.

[24] ARES (2019). ARES Primària.

[25] Evatt M., Ren D., Tuite P., Reynolds C., Hravnak M. (2014). Development and implementation of an educational support process for electronic nursing admission assessment documentation. Medsurg Nurs..

[26] Johnsen L., Karen-Leigh E., Jo-Ann G. (2018). A systematic literature review of accuracy in nursing care plans and using standardized nursing language. Collegian.

[27] Castellà-Creus M., Delgado-Hito P., Casanovas-Cuellar C., Tàpia-Pérez M., Juvé-Udina M.E. (2019). Barriers and facilitators involved in standardized care plan individualisation process in acute hospitalization wards: A grounded theory approach. J. Clin. Nurs..

[28] Johnson R., Burke A.J., Onwuegbuzie A.J., Turner L.A. (2007). Toward a definition of mixed methods research. J. Mix. Methods Res..

[29] Schoonenboom J., Johnson R.B. (2017). How to Construct a Mixed Methods Research Desing. Koln. Z. Soziologie Sozialpsychol..

[30] Greene J.C. (2007). Mixed Methods in Social Inquiry.

[31] CatSalut Regió Sanitaria de Girona. Coneix la RS Girona. CatSalut. Servei Català de la Salut. https://catsalut.gencat.cat/ca/coneix-catsalut/catsalut-territori/girona/.

[32] Caelli K., Ray L., Mill J. (2003). ‘Clear as mud’: Toward greater clarity in generic qualitative research. Int. J. Qual. Methods.

[33] Taylor S.J., Bogdan R., De Vault M. (2015). Introduction to Qualitative Research Methods: A Guidebook and Resource.

[34] Kahlke R.M. (2014). Generic Qualitative Approaches: Pitfalls and Benefits of Methodological Mixology. Int. J. Qual. Methods.

[35] Patton M.Q. (2002). Qualitative Research and Evaluation Methods.

[36] Berenguera-Ossó A., Fernández De Sanmamed Santos M.J., Pons-Vigués M., Pujol-Ribera E., Rodríguez A.D., Saura Sanjaume S. (2014). Escuchar, Observar y Comprender. Recuperando la Narrativa en las Ciencias de la Salud.

[37] Krippendorf (2004). Content Analysis: An Introduction to Its Methodology.

[38] Bengtsson M. (2016). How to plan and perform a qualitative study using content analysis. Nurse Plus Open.

[39] Krueger R.A. (2020). Focus Groups: A Practical Guide for Applied Research.

[40] Alaseeri R., Rajab A., Banakhar M. (2021). Do Personal Differences and Organizational Factors Influence Nurses’ Decision Making? A Qualitative Study. Nurs. Rep..

[41] Chan G.K., Bitton J.R., Allgeyer R.L., Elliott D., Hudson L.R., Moulton Burwell P. (2021). The Impact of COVID-19 on the Nursing Workforce: A National Overview. Online J. Issues Nurs..

[42] Kisley S., Wyder M., Dietrich J., Robinson G., Siskin D., Crompton D. (2016). Motivational aftercare planning to better care: Applying the principales of advanced directives and motivational interviewing to discharge planning for people with mental illness. Int. J. Ment. Health Nurs..

[43] Platt L., Melendez-Torres G.J., O’Donnell A., Bradley J., Newbury-Birch D., Kaner E., Ashton C. (2016). How effective are brief interventions in reducing alcohol consumption: Do the setting, practitioner group and content matter? Findings from a systematic review and metaregression analysis. BMJ Open.

[44] Lukaschek K., Schneider N., Schelle M., Kirk U.B., Eriksson T., Kunnamo I., Rochfort A., Collins C., Gensichen J. (2019). Applicability of Motivational Interviewing for Chronic Disease Management in Primary Care Following a Web-Based E-Learning Course: Cross-Sectional Study. JMIR Ment. Health.

[45] Zoffmann V., Hörnsten Å., Storbækken S., Graue M., Rasmussen B., Wahl A., Kirkevold M. (2016). Translating Person-Centered Care into Practice: A Comparative Analysis of Motivational Interviewing, Illness-Integration Support, and Guided Self-Determination. Patient Educ. Couns..

[46] Van Wilder L., Pype P., Mertens F., Rammant E., Clays E., Devleesschauwer B., Boeckxstaens P., De Smedt D. (2021). Living with a chronic disease: Insights from patients with a low socioeconomic status. BMC Fam. Pract..

[47] Iovino P., Vellone E., Cedrone N., Riegel B. (2023). A Middle-Range Theory of Social Isolation in Chronic Illness. Int. J. Environ. Res. Public Health.

[48] Wu H., Lin W., Li Y. (2023). Health education in the management of chronic diseases among the elderly in the community with the assistance of a Mask R-CNN model. Am. J. Transl. Res..

[49] Fernandes J.B., Teixeira F., Godinho C. (2022). Personalized Care and Treatment Compliance in Chronic Conditions. J. Pers. Med..

[50] Principales Problemas Crónicos de Salud, Porcentaje de Población de 15 y Más Años que Padece Determinados Problemas Crónicos, Registrado en Atención Primaria, por Comunidad Autónoma. https://www.sanidad.gob.es/estadEstudios/sanidadDatos/tablas/tabla5.htm.

[51] Cabo-Meseguer A., Cerda-Olmedo G., Trillo-Mata J.L. (2017). Fibromyalgia: Prevalence, epidemiologic profiles and economic costs. Med. Clin..

[52] Green J., Jester R., McKinley R., Pooler A. (2014). The impact of chronic venous leg ulcers: A systematic review. J. Wound Care.

[53] Ministerio de Sanidad, Servicios Sociales e Igualdad (2015). Estrategia de Seguridad del Paciente del Sistema Nacional de Salud 2015–2020.

[54] Snyder R.J., Jensen J., Applewhite A.J., Couch K., Joseph W.S., Lanti-Ii J.C., Serena T.E. (2019). A Standardized Approach to Evaluating Lower Extremity Chronic Wounds Using a Checklist. Wounds.

[55] Desmedt M., Petrovic M., Bergs J., Vandijck D., Vrijhoef H., Hellings J., Vermeir P., Cool L., Dessers E. (2017). Seen through the patients’ eyes: Safety of chronic illness care. Int. J. Qual. Health Care.

[56] Wears R.L. (2015). Standardisation and its discontents. Cogn. Technol. Work.

[57] De Groot K., De Veer A.J.E., Paans W., Francke A.L. (2020). Use of electronic health records and standardized terminologies: A nationwide survey of nursing staff experiences. Int. J. Nurs. Stud..

[58] Nicklaus J., Kusser J., Zessin J., Amaya M. (2015). Transforming education for electronic health record implementation. J. Contin. Educ. Nurs..

[59] Østensen E., Hardiker N.R., Hellesø R. (2022). Facilitating the Implementation of Standardized Care Plans in Municipal Healthcare. Nursing.

[60] Janssen P.P.M., De Jonge J., Bakker A.B. (1999). Specific determinants of intrinsic work motivation, burnout, and turnover intentions: A study among nurses. J. Adv. Nurs..

[61] World Health Organization (2015). WHO Global Strategy on Integrated People-Centered Health Services 2016–2026.

[62] Hong Q.N., Fàbregues S., Bartlett G., Boardman F., Cargo M., Dagenais P., Gagnon M.P., Griffiths F., Nicolau B., O’Cathain A. (2018). The Mixed Methods Appraisal Tool (MMAT) version 2018 for information professionals and researchers. Educ. Inf..

