# Supplementary material for: Assessment of Standardized Care Plans for People with Chronic Diseases in Primary Care Settings
Source: Nurs Rep. 2024 Mar 29;14(2):801–15. doi: 10.3390/nursrep14020062 (PMC11036219; doi:10.3390/nursrep14020062)
Supplement: Supplementary file 1 [file nursrep-14-00062-s001.zip › nursrep-2877260-supplementary.pdf]

nursing reports

MDPI

Supplementary files

Table S1. Semi-structured ARES-AP focus group guide

**Could you tell us what you think of the ARES-AP programme?**
Could you describe the main advantages of this programme? And the main barriers?
What do you think of the different ARES-AP plans proposed for the care of people with chronic health conditions?
What motivates nurses to work with the ARES-AP plans?
What improvements do you think could be made to the ARES-AP programme?
